# Association of multiple glycemic parameters at intensive care unit admission with mortality and clinical outcomes in critically ill patients

**DOI:** 10.1038/s41598-019-55080-3

**Published:** 2019-12-06

**Authors:** Priscila Bellaver, Ariell F. Schaeffer, Diego P. Dullius, Marina V. Viana, Cristiane B. Leitão, Tatiana H. Rech

**Affiliations:** 1 Graduate Program in Medical Sciences: Endocrinology, Universidade Federal do Rio Grande do Sul (UFRGS), Porto Alegre, RS Brazil; 2 Department of Internal Medicine, Hospital de Clínicas de Porto Alegre, Porto Alegre, RS Brazil; 3 School of Medicine, Universidade Federal do Rio Grande do Sul (UFRGS), Porto Alegre, RS Brazil; 4 Department of Surgery, Hospital de Clínicas de Porto Alegre, Porto Alegre, RS Brazil; 5 Intensive Care Unit, Hospital de Clínicas de Porto Alegre, Porto Alegre, RS Brazil; 6 Endocrine Division, Hospital de Clínicas de Porto Alegre, Porto Alegre, RS Brazil

**Keywords:** Endocrinology, Endocrine system and metabolic diseases, Diabetes

## Abstract

The aim of the present study was to investigate the association of multiple glycemic parameters at intensive care unit (ICU) admission with outcomes in critically ill patients. Critically ill adults admitted to ICU were included prospectively in the study and followed for 180 days until hospital discharge or death. Patients were assessed for glycemic gap, hypoglycemia, hyperglycemia, glycemic variability, and stress hyperglycemia ratio (SHR). A total of 542 patients were enrolled (30% with preexisting diabetes). Patients with glycemic gap >80 mg/dL had increased need for renal replacement therapy (RRT; 37.7% vs. 23.7%, p = 0.025) and shock incidence (54.7% vs. 37.4%, p = 0.014). Hypoglycemia was associated with increased mortality (54.8% vs. 35.8%, p = 0.004), need for RRT (45.1% vs. 22.3%, p < 0.001), mechanical ventilation (MV; 72.6% vs. 57.5%, p = 0.024), and shock incidence (62.9% vs. 35.8%, p < 0.001). Hyperglycemia increased mortality (44.3% vs. 34.9%, p = 0.031). Glycemic variability >40 mg/dL was associated with increased need for RRT (28.3% vs. 14.4%, p = 0.002) and shock incidence (41.4% vs.31.2%, p = 0.039). In this mixed sample of critically ill subjects, including patients with and without preexisting diabetes, glycemic gap, glycemic variability, and SHR were associated with worse outcomes, but not with mortality. Hypoglycemia and hyperglycemia were independently associated with increased mortality.

## Introduction

In critically ill patients, hyperglycemia is an adaptive metabolic response to acute stress^1,2^. Optimal glycemic targets in the ICU setting are controversial and seem to be related to previous patient metabolic status^3–9^. As glycated hemoglobin (HbA1c) is not affected by the onset of acute illness, it can be used to estimate chronic glycemic control in critically ill patients^10^. In the intensive care setting, patients with higher HbA1c have higher mortality^11,12^. However, patients with poor chronic glycemic control have worse outcomes when treated with intensive glycemic control^13^, suggesting that chronic hyperglycemia might be able to generate cellular mechanisms that protect against damage mediated by acute hyperglycemia during critical illness^14^. Therefore, the question is more complex than selecting the “optimal glycemic target”, as the interplay between multiple domains of glycemic control might be more relevant. In this sense, glycemic variability, which reflects the magnitude of glycemic excursions during the day, has been associated with unfavorable outcomes in critical illness, including higher mortality^15,16^.

In addition, to separate preexisting hyperglycemia from stress-induced hyperglycemia (elevated blood glucose that reverts to normal after disease and inflammation subsides), the glycemic gap and the stress hyperglycemia ratio (SHR) have been proposed as predictors of adverse outcomes in the ICU. The glycemic gap, defined as the difference between blood glucose at ICU admission and the estimated mean blood glucose derived from HbA1c values, is associated with worse prognosis in specific populations of critically ill patients, such as patients with acute myocardial infarction, community-acquired pneumonia, and hepatic abscess^17–19^. Moreover, a glycemic gap above 80 mg/dL is associated with higher hospital mortality in critically ill patients with DM^20^, but its value as a prognostic tool in mixed medical-surgical sample of critically ill subjects is unknown. The SHR is calculated by dividing the blood glucose on admission by the estimated average glucose derived from HbA1c. Studies suggest that an SHR > 1.1 is a better predictor for worse outcomes in critical illness than the absolute mean blood glucose^21^.

Within this context, the aim of the present study is to investigate the association of multiple glycemic parameters (glycemic gap, hypoglycemia, hyperglycemia, glycemic variability, and SHR) at ICU admission with clinical outcomes in critically ill patients with and without diabetes. The primary endpoint was mortality. Secondary endpoints were need for renal replacement therapy (RRT), incidence of shock, need for mechanical ventilation (MV), time spent on MV, length of stay (LOS) in the hospital, LOS in the ICU, and need for ICU readmission.

## Results

### Study population characteristics

A total of 542 consecutive patients admitted to the ICU were included in the study. Their main characteristics are summarized in Supplementary Table S1. Briefly, 52.5% were male, and the mean age was 59 ± 15 years; 42.4% were admitted to ICU from the emergency department. ICU admissions were for medical reasons in 84.3% and for surgical reasons in 15.7%, with acute respiratory failure as the leading cause (23.6%). The most common primary coexisting condition was hypertension (54%), followed by DM (30%). Mean serum glucose at admission was higher in nonsurvivors than in survivors (146 ± 73 vs. 132 ± 60 mg/dL, p = 0.023), but HbA1c was not (5.8 ± 1.7 vs. 5.5 ± 1.4% p = 0.058). The overall mortality rate was 38.2%. When analyzing patients with DM separately, the mortality rate was similar regardless of preexisting DM (43% vs. 38.2%; p = 0.193). In patients with DM (n = 163; n = 4 type 1 DM; n = 159 type 2 DM), mean blood glucose and HbA1c were similar in survivors and nonsurvivors (mean blood glucose: 166 ± 87 vs. 187 ± 96, p = 0.16; HbA1c 6.8 ± 1.8% vs. 6.9 ± 2.4, p = 0.705).

### Glycemic parameters and mortality

**Figure 1:**
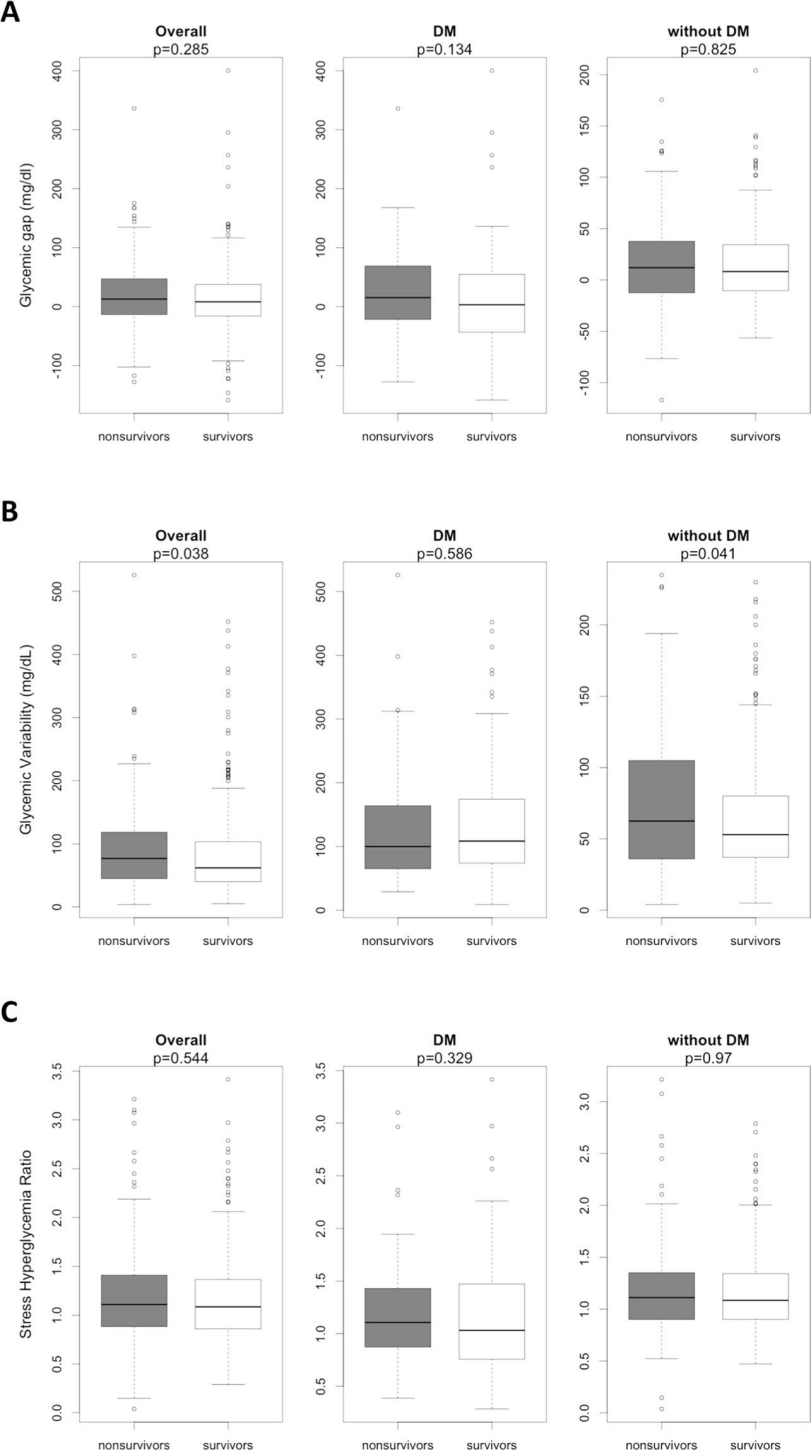
Box plot demonstrating the effect of glycemic gap (**A**), glycemic variability (**B**), and stress hyperglycemia ratio (**C**) on mortality in overall population, in patients with DM (diabetes mellitus), and in patients without DM. Values are median and interquartile range; dots represent outliers. Figures were constructed using R version 3.5.2 (The R Foundation, version 3.5.1, 2018).

The glycemic gap varied widely, from −159 to 400, with a median value of 11 (−15 to 42) mg/dL in the overall population and no difference between survivors and nonsurvivors was observed (Fig. 1A). When analyzing patients with DM separately, the glycemic gap was 13 (−37 to 61) mg/dL, and no differences were observed between survivors and nonsurvivors. Similarly, in patients without preexisting DM, the glycemic gap was not different in survivors and nonsurvivors. When stratifying patients by a glycemic gap cutoff of 80 mg/dL, a trend toward higher mortality was observed in subjects with wider glycemic gaps (49% vs. 37%; p = 0.089). Data related to glycemic gap cutoff of >40 mg/dL and >60 mg/dL are summarized in Supplementary Table S2.

More patients who died had hypoglycemia (54.8% vs. 35.8%, p = 0.004) and hyperglycemia (44.3% vs. 34.9%, p = 0.031) compared to survivors.

In the entire sample, mean glycemic variability was 67 (41 to 112) mg/dL, with higher values in nonsurvivors (Fig. 1B). This difference is not demonstrated in patients with preexisting DM, but is clearly seem in patients without DM. When patients with a glycemic variability cutoff of 40 mg/dL were analyzed in separate, no difference in mortality was observed in subjects with higher glycemic amplitudes (34% vs. 39%; p = 0.228). Data related to variability >60 mg/dL and >80 mg/dL are summarized in Supplementary Table S3. Overall SHR was 1.1 (±0.5), with no difference between survivors and nonsurvivors (Fig. 1C), independently or preexisting DM or not.

### Glycemic parameters and other outcomes

When comparing patients with glycemic gap above and below 80 mg/dL, the group with higher values had increased need for RRT and a higher incidence of shock (Table 1).

**Table 1:** Effects of stress-induced hyperglycemia on clinical outcomes.

Outcomes	Glycemic gap		p	SHR		p
	<80 mg/dL (n = 489)	>80 mg/dL (n = 53)		<1.1 (n = 267)	>1.1 (n = 275)	
Mortality (n, %)	181 (37)	26 (49)	0.087	96 (36)	111 (40)	0.247
Need for RRT (n, %) Shock incidence (n, %)	116 (23.7) 183 (37.4)	20 (37.7) 29 (54.7)	**0.025** **0.014**	62 (23) 96 (36)	74 (27) 116 (42)	0.287 0.112
Need for MV (n, %)	286 (58.4)	36 (67.9)	0.184	141 (52.8)	181 (65.8)	**0.001**
Time on MV (days)	4 (1 to 8)	4.5 (2 to 8)	0.117	3 (1 to 8)	2 (4 to 8.5)	**<0.001**
LOS, hospital (days)	20 (10 to 35)	20 (10 to 34.7)	0.585	19 (10.7 to 32.5)	21 (9 to 38)	0.263
LOS, ICU (days)	7 (3 to 12)	7.5 (4.25 to 11)	0.126	6 (3 to 11)	8 (4 to 12)	**0.005**
ICU readmission (n, %)	62 (12.6)	6 (11.3)	0.770	30 (11.2)	38 (13.8)	0.339

SHR: stress hyperglycemia ratio; RRT: renal replacement therapy; MV: mechanical ventilation; LOS: length of stay; ICU: intensive care unit. Glycemic gap was calculated by the difference between the serum glucose at ICU admission and the estimated mean blood glucose derived from HbA1c. SHR was defined by the ratio between serum glucose at admission and the estimated mean blood glucose derived from HbA1c. Values are mean ± SD or median and interquartile range.

The presence of hypoglycemia within the first 24 hours of ICU admission was associated with increased need for RRT, increased need for MV, and higher incidence of shock. Length of hospital stay was shorter in this population. The presence of hyperglycemia on admission was associated with increased need for MV. Glycemic variability during the first 24 hours of admission was associated with an increased need for RRT and a higher incidence of shock. These results are summarized in Table 2 and Supplementary Table S3. SHR > 1.1 was associated with greater need for MV, but with a shorter time spent on MV and longer ICU stay (Table 1). Interestingly, no glycemic control parameters were associated with the need for ICU readmission (Tables 1 and 2).

**Table 2:** Effects of hypoglycemia, hyperglycemia, and glycemic variability on clinical outcomes.

Outcomes	Hypoglycemia		p	Hyperglycemia		p	Glycemic variability		p
	No	Yes		No	Yes		<40 mg/dL	>40 mg/dL	
	(n = 480)	(n = 62)		(n = 350)	(n = 192)		(n = 125)	(n = 417)	
Mortality (n, %)	172 (35.8)	34 (54.8)	**0.004**	122 (34.9)	85 (44.3)	0.031	42 (33.6)	165 (39.5)	0.228
Need for RRT (n, %)	107 (22.3)	28 (45.1)	**<0.001**	81 (23.1)	55 (28.6)	0.158	18 (14.4)	118 (28.3)	**0.002**
Shock incidence (n, %)	172 (35.8)	39 (62.9)	**<0.001**	128 (36.6)	84 (43.7)	0.101	39 (31.2)	173 (41.4)	**0.039**
Need for MV (n, %)	276 (57.5)	45 (72.6)	**0.024**	195 (55.7)	127 (66.1)	**0.018**	64 (51.2)	258 (61.8)	**0.033**
Time on MV (days)	4 (2 to 8)	2 (1 to 6)	0.365	4 (1 to 8)	4 (2 to 7.7)	**0.014**	4.5 (1.2 to 9)	4 (1 to 8)	0.17
LOS, hospital (days)	21 (11 to 36)	14 (5 to 30)	**0.024**	21 (11 to 37)	17.5 (8 to 33.5)	0.498	22 (12–35)	19 (9 to 36)	0.575
LOS, ICU (days)	7 (4 to 12)	5 (2 to 8.5)	0.117	7 (3 to 12)	7 (4 to 11)	0.183	8 (4 to 12.7)	7 (3 to 11)	0.898
ICU readmission (n, %)	64 (13.3)	4 (6.5%)	0.123	41 (11.7)	27 (14)	0.43	14 (11.2)	54 (12.9)	0.604

RRT: renal replacement therapy; MV: mechanical ventilation; LOS: length of stay; ICU: intensive care unit. Hypoglycemia was defined as any serum or capillary glucose <70 mg/dL during the first ICU day. Hyperglycemia was defined as any serum glucose >140 mg/dL at ICU admission. Glycemic variability was calculated as the absolute difference in capillary blood glucose during the first ICU day. Values are mean ± SD or median and interquartile range.

### Magnitude of the association between glycemic parameters and outcomes

**Figure 2:**
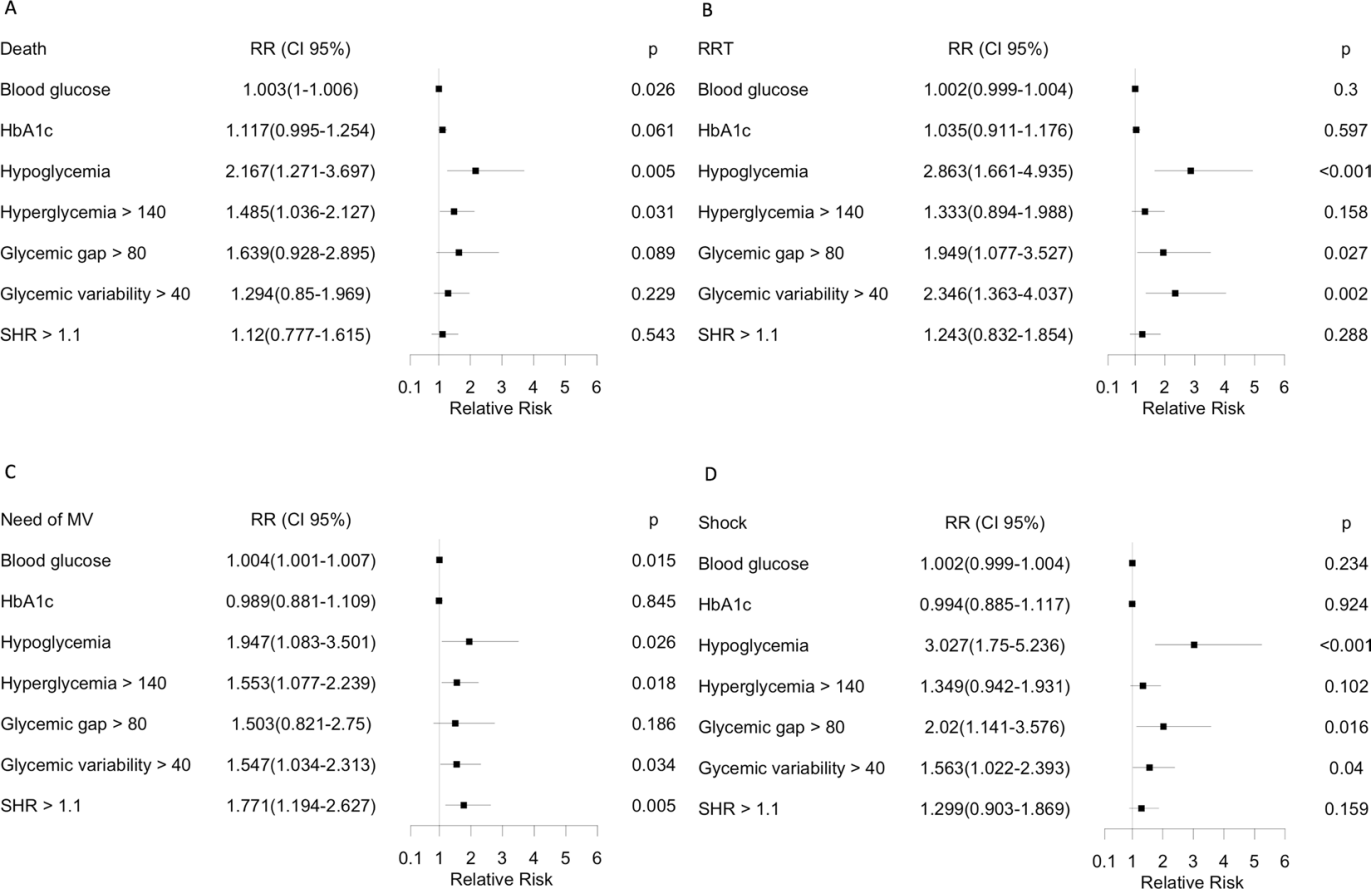
Relative risks for outcomes according to each glycemic parameter. (**A**) Mortality. (**B**) Need for renal replacement therapy (RRT). (**C**) Incidence of shock. (**D**) Need for mechanical ventilation (MV). HbA1c: glycated hemoglobin. Hypoglycemia was defined as any serum or capillary glucose <70 mg/dL during the first ICU day. Hyperglycemia was defined as any serum glucose >140 mg/dL at ICU admission. Glycemic gap was calculated by the difference between serum glucose at ICU admission and the estimated mean blood glucose derived from HbA1c. Glycemic variability was calculated as the absolute difference in capillary blood glucose during the first ICU day. SHR (stress of hyperglycemia ratio) was defined by the ratio between serum glucose at admission and the estimated mean blood glucose derived from HbA1c.Values are point estimates with 95% confidence intervals. Figures were constructed using R version 3.5.2 (The R Foundation, version 3.5.1, 2018).

Figure 2 presents the relative risks (RR) for outcomes according to each glycemic parameter. Glycemic gap >80 mg/dL was associated with an increased need for RRT and significantly increased the incidence of shock.

Mean blood glucose increased risk of death, but HbA1c did not. Hypoglycemia was the parameter with the highest RR for mortality, followed by hyperglycemia and glycemic variability. Hypoglycemia also yielded the highest RR for the need for RRT. Glycemic variability was also associated with an increased need for RRT. Besides glycemic gap >80 mg/dL, two other glycemic parameters significantly increased the incidence of shock: hypoglycemia and glycemic variability. Regarding the need for MV, hypoglycemia was associated with the highest risk, followed by hyperglycemia. Glycemic variability also increased the need for MV. No glycemic parameter was associated with risk of ICU readmission, as shown in Supplementary Fig. S1.

**Figure 3:**
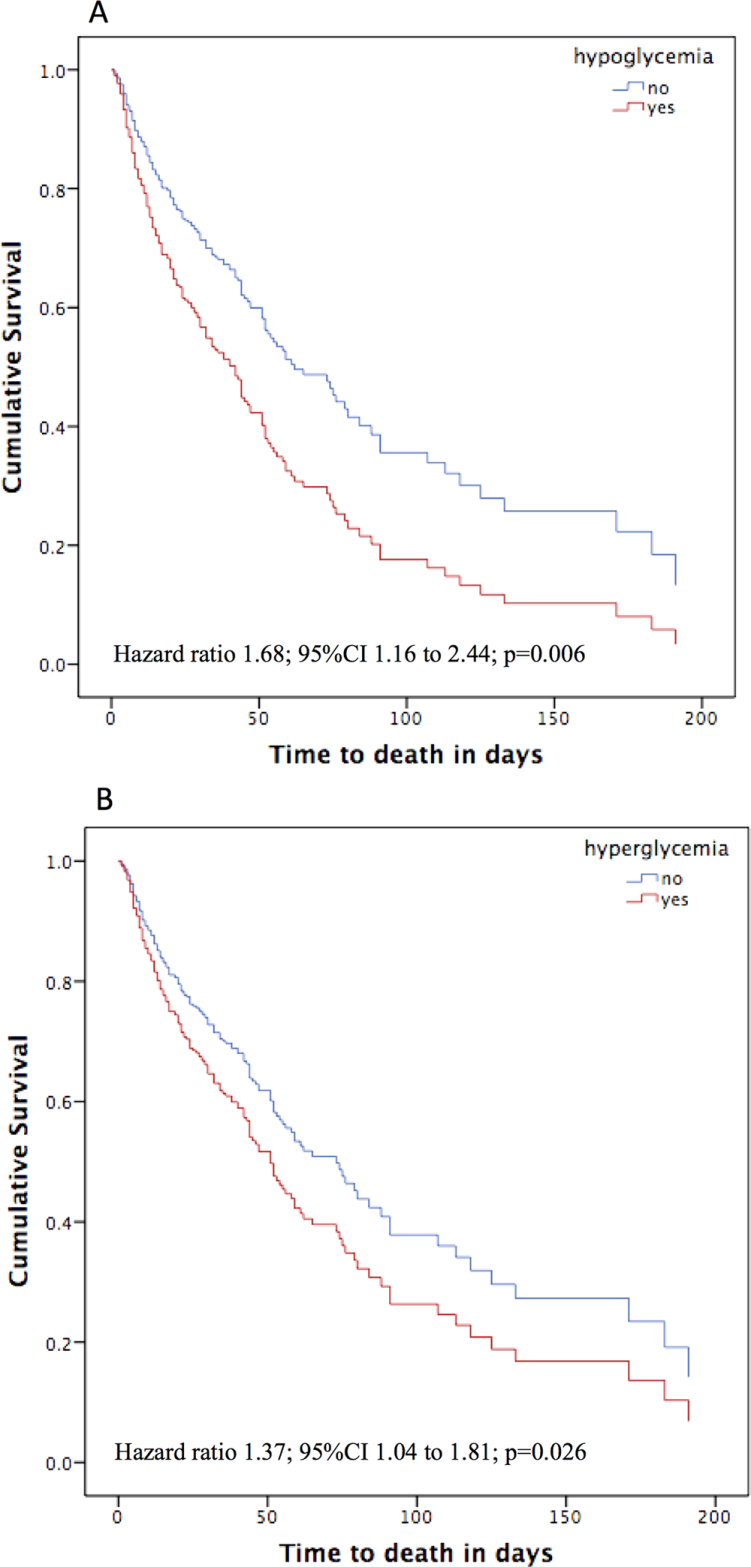
Cumulative survival at 180 days stratified by presence of abnormal blood glucose at ICU admission. (**A**) Hypoglycemia (defined as any serum or capillary glucose measurement <70 mg/dL during the first ICU day). (**B**) Hyperglycemia (defined as any serum glucose measurement >140 mg/dL at ICU admission). Hazard ratios are adjusted for SAPS 3 (Simplified Acute Physiology III) score. Figures were constructed using SPSS 20.0 (Chicago, IL, USA).

Cox regression multivariate analysis with adjustment for SAPS 3 was calculated for the glycemic parameters associated with mortality in the univariate models. Three glycemic parameters were associated with increased mortality in the univariate analysis: hypoglycemia (Table 2), hyperglycemia (Table 2) and glucose variability (cutoff >60 mg/dL and >80 mg/dL, Supplementary Table S3). Hypoglycemia and hyperglycemia remained associated with mortality after adjustments (HR 1.68; 95%CI 1.16 to 2.44, p = 0.006 and HR 1.37; 95%CI 1.04 to 1.81, p = 0.026, for hypoglycemia and hyperglycemia, respectively) (Fig. 3A,B), but glycemic variability did not (HR 1.14; 95%CI 0.86 to 1.51, p = 0.366 for variability >60 mg/dL and HR 1.25; 95%CI 0.95 to 1.64, p = 0.104 for variability >80 mg/dL).

## Discussion

In this sample of unselected, prospectively followed critically ill patients, including those with and without preexisting diabetes, multiple glycemic parameters at ICU admission were associated with worse outcomes. Higher glycemic gap, a marker of stress-induced hyperglycemia, increased the need for RRT and incidence of shock, but had not impact on mortality. The presence of hypoglycemia in the first 24 hours of ICU admission was associated with the highest RR for worse outcomes, including increased mortality; a single episode of hypoglycemia doubled the risk of death and increased the incidence of shock and the need for RRT threefold. Additionally, hyperglycemia at ICU admission increased the risk of mortality by approximately 50%. Both hypoglycemia and hyperglycemia were independently associated with mortality.

The association between hyperglycemia and mortality in acutely ill patients, especially in those without diabetes, is well documented in the literature^22,23^. However, it is still not clear if hyperglycemia is a marker of disease severity or a determinant of prognosis by itself. In our study, the fact that hyperglycemia was independently associated with mortality corroborates the later. Furthermore, there is physiological plausibility to justify how high glucose levels contribute to worse outcomes, for instance, the deleterious effects of hyperglycemia on immune system function and the increased proteolysis producing lean tissue breakdown^24^. These two mechanisms might contribute to infectious complications^25^ and ICU-acquired muscular weakness^26^ respectively.

Glycemic gap and SHR are markers of acute-stress hyperglycemia, as they are able to separate the effect of acute, critical illness-associated variations in blood glucose from the previous metabolic state. The present study was the first to analyze the role of glycemic gap and SHR in a general medical-surgical population of ICU patients. Unexpectedly, no increase in mortality was demonstrated in patients with high glycemic gaps or high SHR, even when patients with DM were analyzed separately, which stands in contrast to the findings of previous small studies of glycemic gap in very specific populations critically ill patients with diabetes^17–19^. However, a high glycemic gap doubled the risk of shock and the need for RRT and SHR increased risk of the need for MV and the time spent on MV. The main differences in our study are a larger sample size, the inclusion of a mixed sample of surgical and medical critically ill patients, with and without DM, and with high disease severity scores. Therefore, our sample represents a more heterogeneous population, which increases the clinical applicability of our findings.

Some studies suggest that glucose variability during the 24 hours is a better predictor of outcomes in critical illness than mean blood glucose^15,16,27,28^. However, our results show that glycemic variability measured in the first 24 hours of admission was associated with worse outcomes, increased need for RRT and MV, and higher incidence of shock, but only hypoglycemia and hyperglycemia were associated with increased mortality after adjustment for disease severity. This contrasting result with the literature might be explained by the metric of glucose variability used in our study (difference of maximum and minimum glucose values). Although, we have to highlight that the best indicator for glucose variability quantification is unsettled^29^ and that a retrospective analysis of the Leuven studies^8,9^ showed that increased daily glucose amplitude was associated with increased mortality^30^. Moreover, glycemic variability was not associated with higher risk of mortality in patients with DM, corroborating the idea that patients exposed to chronic hyperglycemia are adapted to glycemic excursions, possibly developing protective cellular mechanisms against wide blood glucose variations during the course of a critical illness^14^. In our study, the mortality of patients with DM was similar to that of patients without DM, in agreement with the concept of the “diabetic paradox” in the ICU—i.e., the finding that DM is not independently associated with increased risk of mortality in heterogeneous populations of critically ill patients^31^.

This study has limitations. First, glucose monitoring was not continuous, which raises the possibility that some extreme glucose values may have gone unrecorded. However, this is a conservative bias that might have decreased differences in outcomes, further corroborating our findings. Furthermore, glucometers are still largely used because they are practical and low cost. Second, accurate information on diet (including the amount of calories consumed) and on insulin doses administered was not recorded, thus precluding conclusions regarding the influence of carbohydrate intake or insulin therapy on outcomes. However, the association of at least one of the outcomes evaluated herein, hypoglycemia, seems to be independent from insulin use, as suggested by the results of the NICE-SUGAR study^32^. Third, the absence of data on diabetes duration and the low absolute number of patients with type 1 DM prevent any conclusion regarding different underlying pathogenic mechanisms between type 1 and type 2 DM and long term complications over the outcomes.

The main strength of this study is the prospective cohort design, specifically selected to evaluate multiple glycemic parameters simultaneously in a large unselected population of critically ill patients and their associations with clinical outcomes and mortality. Our findings reinforce that, besides mean blood glucose seems to be the most important predictor of outcomes, several other domains of glycemic control are relevant in critical illness. This might be especially important for research in glycemic control, as future trials should evaluate not only glycemic targets, but rather multiple glycemic parameters at the same time.

In summary, in this mixed medical-surgical sample of critically ill subjects, including patients with and without previous diagnosis of diabetes, glycemic gap was associated with worse clinical outcomes, but had no impact on mortality. Hypoglycemia and hyperglycemia were independently associated with increased mortality and influenced other outcomes, such as incidence of shock and need for RRT and MV. Similarly to glycemic gap, glycemic variability and SHR also negatively affected outcomes, with no impact on mortality. It is common sense that optimal glycemic control should be pursued to reduce the risk of unfavorable outcomes. Then, further research is needed to personalize glycemic control targets in critically ill patients, focusing on a broader view of glucose dysregulation based in multiple parameters rather than in a single, in an effort to reduce the risks of iatrogenic adverse events.

## Methods

### Study population

This is a prospective cohort study. The study protocol was approved by the ethics committee at Hospital de Clínicas de Porto Alegre (project number 17-0386). Informed consent was obtained from patients or their legal representatives. From September 2017 to February 2018, critically ill adults (age >18 years) admitted to the ICU were prospectively included in the study. The exclusion criteria were pregnancy, diabetic ketoacidosis, hyperosmolar hyperglycemic state, sickle cell anemia, and other hemoglobinopathies. Blood samples were collected at study entry from all patients for random serum blood glucose and HbA1c quantifications, and clinical and laboratory data were recorded for all patients. Simplified Acute Physiology Score 3 (SAPS 3), ranging theoretically from a minimum of 0 points to a maximum of 271 points, with higher scores denoting higher severity^33^, was used to score disease severity.

Hyperglycemia was defined according to the American Diabetes Association (ADA) proposed threshold for in-hospital hyperglycemia as any blood glucose measurement >140 mg/dL^34–36^ at ICU admission. Moderate hypoglycemia was any blood or capillary glucose <70 mg/dL (ADA definition and same level from the NICE-SUGAR study)^32,34^ and serious hypoglycemia was <54 mg/dL (ADA definition)^34^ during the first day in the ICU. Glucose variability was calculated as the absolute difference in capillary blood glucose during the first day in the ICU^15,16^. In order to separate the effects of a chronically altered metabolic state from those of acute stress hyperglycemia, the glycemic gap and SHR were evaluated. The glycemic gap was calculated by the difference between the ICU admission serum blood glucose and the estimated mean blood glucose (serum blood glucose on ICU admission − estimated mean blood glucose)^17,18^. HbA1c values were used to calculate the estimated mean blood glucose, using the following formula: (28.7 × HbA1c) − 46.7 mg/dL (30). The cutoff value of 80 mg/dL for glycemic gap was based on Liau *et al*.^20^. SHR was defined by the ratio between serum blood glucose at admission and the estimated mean blood glucose and the cutoff value of 1.1 based on Roberts *et al*.^21^. Diabetes was defined on the basis of previous diagnosis or when HbA1c was ≥6.5%^34^. HbA1c quantifications were measured for all patients at ICU admission. Previous diagnosis of diabetes was assessed by two researchers (P.B. and A.S) by electronic chart review (hospital and ICU admission notes, previous use of antihyperglycemic agents or insulin, and outpatient visits if needed).

The outcomes of interest were adjudicated by two unblinded researchers (P.B. and A.S) and included the following: mortality (primary endpoint) and need for RRT, incidence of shock, need for MV, time spent on MV, LOS in the hospital, LOS in the ICU, and need for ICU readmission (secondary endpoints). All patients were followed up for 180 days for survival analysis.

### Biochemical measurements

Blood samples for glucose measurement were collected in tubes with sodium fluoride, centrifuged for 10 min at 3670 rpm, and analyzed by the hexokinase method in a Roche COBAS c702 system (Roche Diagnostics, Mannheim, GE). For HbA1c measurement, blood samples were collected in EDTA tubes, homogenized, and analyzed in BioRAD Variant Turbo II (BioRAD, Hercules, California, USA), processed by HPLC. Glucose values were expressed as mg/dL, and HbA1c values as percentage.

### Statistical analysis

Categorical variables were expressed as percentages. Data were expressed as mean and standard deviation (SD) if normally distributed, or as median and interquartile range otherwise. Groups were compared using Student’s *t*-test, the Mann–Whitney *U* test, or the chi-square test as appropriate. To assess relative risks of variables of interest and outcomes, univariate linear regression or logistic regression models were constructed depending on the characteristics of the outcomes of interest. A multivariate Cox regression analysis with mortality as outcome was used to calculate hazard ratios (HR), with variables adjusted for disease severity using SAPS 3 score. A sample size of 494 patients was calculated considering a power of 95% and an α-error rate of 5% to detect a difference in glycemic gap of 42 mg/dL between survivors and nonsurvivors. There was no missing data. Values were considered statistically significant if p < 0.05. Statistical analyses were conducted in SPSS 20.0 (Chicago, IL, USA) and R version 3.5.2 (The R Foundation, version 3.5.1, 2018).

### Ethical approval

All experiments were performed in accordance with relevant guidelines and regulations. The study protocol was approved by the Ethics Committee at Hospital de Clínicas de Porto Alegre (project number 17-0386).

## Supplementary Material

Supplementary information
