# Supplementary material for: Association of multiple glycemic parameters at intensive care unit admission with mortality and clinical outcomes in critically ill patients
Source: Sci Rep. 2019 Dec 6;9:18498. doi: 10.1038/s41598-019-55080-3 (PMC6897941; doi:10.1038/s41598-019-55080-3)
Supplement: Supplementary file 1 — Supplementary information [file 41598_2019_55080_MOESM1_ESM.pdf]

Association of multiple glycemic parameters at intensive care unit admission with mortality and clinical outcomes in critically ill patients

Priscila Bellaver, MD[1,2], Ariell F. Schaeffer[3], Diego P. Dullius, MD[4], Marina V. Viana, MD, PhD[5], Cristiane B. Leitão, MD, PhD[1,6], Tatiana H. Rech, MD, PhD[1,5,*]

[1] Graduate Program in Medical Sciences: Endocrinology, Universidade Federal do Rio Grande do Sul (UFRGS), Porto Alegre, RS, Brazil;

[2] Department of Internal Medicine, Hospital de Clínicas de Porto Alegre, Porto Alegre, RS, Brazil;

[3] School of Medicine, Universidade Federal do Rio Grande do Sul (UFRGS), Porto Alegre, RS, Brazil;

[4] Department of Surgery, Hospital de Clínicas de Porto Alegre, Porto Alegre, RS, Brazil;

[5] Intensive Care Unit, Hospital de Clínicas de Porto Alegre, Porto Alegre, RS, Brazil;

[6] Endocrine Division, Hospital de Clínicas de Porto Alegre, Porto Alegre, RS, Brazil.

Supplementary Table S1. Baseline characteristics of patients.

Characteristics	Patients (n=542)
Age (years)	59 ± 15
Men (n, %)	285 (52.5)
BMI (kg/m^2)	27.2 ± 6.6
SAPS 3	57 ± 15
Coexisting conditions (n)	3.5 ± 2
Hypertension (n, %)	293 (54)
Diabetes (n, %)	163 (30)
Cancer (n, %)	108 (20)
Chronic kidney disease (n, %)	97 (18)
Ischemic heart disease (n, %)	97 (18)
Heart failure (n %)	76 (14)
COPD (n, %)	65 (12)
Other (n, %)	92 (17)
Reason for ICU admission	
Acute respiratory failure (n, %)	128 (23.6)
Shock (n, %)	122 (22.1)
Neurologic disorder (n, %)	94 (17.3)
Major surgery (n, %)	80 (14.7)
Cardiovascular disorders (n, %)	37 (6.8)
Other (n, %)	82 (15)
Sepsis (n, %)	351 (64.7)
Need for mechanical ventilation (n, %)	322 (59)
Use of vasopressors (n, %)	331 (61.1)
Need for renal replacement therapy (n, %)	136 (25)
Use of glucocorticosteroids (n, %)	231 (42.8)
Nutrition (n, %)	384 (70.8)
Enteral (n, %)	376 (69.4)
Parenteral (n, %)	8 (1.48)
Insulin therapy (n, %)	148 (27.3)
Long-acting insulin (n, %)	21 (3.9)
Short-acting insulin (n, %)	94 (17.3)
Both long and short-acting insulin (n, %)	33 (6.1)
Blood glucose (mg/dL)	137 ± 65
HbA1c (%)	5.6 ± 1.5
Hyperglycemia (n, %)	192 (35.4)
Hypoglycemia <70 mg/dL (n, %)	62 (11.4)
Hypoglycemia <54 mg/dL (n, %)	25 (4.6)
Glycemic variability (mg/dL)	67 (41 to 112)
Glycemic gap (mg/dL)	11.3 (-15.7 to 42)
Stress hyperglycemia ratio	1.1 ± 0.4

BMI: body mass index; SAPS 3: Simplified Acute Physiology III; COPD: chronic obstructive pulmonary disease; ICU: intensive care unit; HbA1c: glycated hemoglobin. Hyperglycemia was defined as any serum glucose >140 mg/dL at ICU admission. Hypoglycemia was defined as any serum or capillary glucose <70 mg/dL during the first ICU day; if <54 mg/dL, it was defined as serious hypoglycemia. Glycemic variability was calculated as the absolute difference in capillary blood glucose during the first ICU day. Glycemic gap was calculated by the difference between the serum glucose at admission and the estimated mean blood glucose derived from HbA1c. Stress hyperglycemia ratio was defined by the ratio between serum glucose at admission and the estimated mean blood glucose derived from HbA1c. Values are mean ± SD or median and interquartile range.

Supplementary Table S2. Effects of glycemic gap >40 mg/dL and >60 mg/dL on clinical outcomes.

Outcomes	Glycemic gap					
	**<40 mg/dL (n = 401)**	**>40 mg/dL (n = 141)**	**p**	**<60 mg/dL (n = 454)**	**>60 mg/dL (n = 88)**	**p**
**Mortality (n, %)**	147 (36.7)	60 (42.6)	0.215	167 (6.8)	40 (45.5)	0.125
**Need for RRT (n, %)**	88 (22)	48 (34)	**0.004**	108 (23.8)	28 (31.8)	0.112
**Shock incidence (n, %)**	145 (36.2)	67 (47.5)	**0.017**	169 (37.2)	43 (49)	**0.041**
**Need for MV (n, %)**	227 (56.6)	95 (67.4)	**0.025**	226 (58.6)	56 (63.6)	0.378
**Time on MV (days)**	1 (0 to 5)	2 (0 to 6)	**0.044**	1 (0 to 6)	1.5 (0 to 6)	0.356
**LOS, hospital (days)**	18 (10 to 34)	22 (11 to 35.5)	0.618	19 (10 to 34)	20.5 (10 to 35)	0.962
**LOS, ICU (days)**	4 (2 to 9)	5 (3 to 9)	0.225	4 (2 to 9)	5 (3 to 9)	0.569
**ICU readmission (n, %)**	45 (11.2)	23(16.3)	0.117	54 (12)	14 (16)	0.330

RRT: renal replacement therapy; MV: mechanical ventilation; LOS: length of stay; ICU: intensive care unit. Glycemic variability was calculated as the absolute difference in capillary blood glucose during the first ICU day. Values are mean ± SD or median and interquartile range.

Supplementary Table S3. Effects of glycemic variability >60 mg/dL and >80 mg/dL on clinical outcomes.

	**Glycemic variability**					
**Outcomes**	**<60 mg/dL (n = 235)**	**>60 mg/dL (n = 307)**	**p**	**<80 mg/dL (n = 313)**	**>80 mg/dL (n = 229)**	**p**
**Mortality (n, %)**	78 (32.7)	129 (42)	**0.036**	107 (34.1)	100 (43.6)	**0.025**
**Need for RRT (n, %)**	39 (16.6)	97 (31.6)	**<0.001**	58 (18.5)	78 (34)	**<0.001**
**Shock incidence (n, %)**	80 (34)	132 (42.9)	**0.034**	108 (34.5)	104 (45.4)	**0.010**
**Need for MV (n, %)**	128 (54.4)	194 (63.1)	**0.040**	175 (55.9)	147 (64.2)	0.052
**Time on MV (days)**	4 (1 to 8)	4 (2 to 8)	0.077	4 (2 to 8.7)	4 (1 to 7)	0.176
**LOS, hospital (days)**	20 (11.5 to 36)	20 (9 to 35.2)	1	21 (10.2 to 38)	20 (9 to 34)	0.658
**LOS, ICU (days)**	7 (3.5 to 12)	7 (3 to 11)	0.602	7 (4 to 12)	6 (3 to 11)	0.569
**ICU readmission (n, %)**	28 (11.9)	40 (13)	0.698	42 (13.4)	26 (11.3)	0.473

RRT: renal replacement therapy; MV: mechanical ventilation; LOS: length of stay; ICU: intensive care unit. Glycemic variability was calculated as the absolute difference in capillary blood glucose during the first ICU day. Values are mean ± SD or median and interquartile range.

Readmission	RR (CI 95%)	p
Blood glucose	1(0.996-1.004)	0.909
HbA1c	0.946(0.789-1.135)	0.552
Hypoglycemia	0.447(0.157-1.274)	0.132
Hyperglycemia > 140	1.233(0.732-2.077)	0.43
Glycemic gap > 80	0.879(0.361-2.142)	0.777
Glycemic variability > 40	1.179(0.631-2.204)	0.605
SHR > 1.1	1.141(0.676-1.925)	0.621

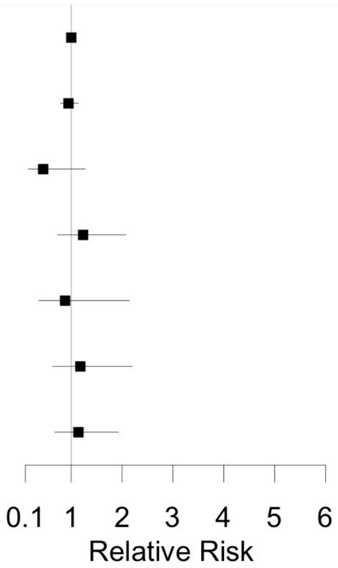

Supplementary Figure S1. Relative risks for ICU readmission according to each glycemic parameter. HbA1c: glycated hemoglobin. Hypoglycemia was defined as any serum or capillary glucose <70 mg/dL during the first ICU day. Hyperglycemia was defined as any serum glucose >140 mg/dL at ICU admission. Glycemic gap was calculated as the difference between the serum glucose at ICU admission and the estimated mean blood glucose derived from HbA1c. Glycemic variability was calculated as the absolute difference in capillary blood glucose during the first ICU day. SHR (stress of hyperglycemia ratio) was defined by the ratio between serum glucose at admission and the estimated mean blood glucose derived from HbA1c. Values are point estimates with 95% confidence intervals.
